# *Swertia mussotii* prevents high-fat diet-induced non-alcoholic fatty liver disease in rats by inhibiting expression the TLR4/MyD88 and the phosphorylation of NF-κB

**DOI:** 10.1080/13880209.2022.2127153

**Published:** 2022-10-07

**Authors:** Ming Dong Si, Meng Wu, Xi Zhen Cheng, Zhi Hong Ma, Yu Guang Zheng, Jing Li, Si Li, Yong Xing Song, Donglai Ma

**Affiliations:** a School of Pharmacy, Hebei University of Chinese Medicine, Shijiazhuang, China; b School of Basic Medicine, Hebei University of Chinese Medicine, Shijiazhuang, China; c Traditional Chinese Medicine Processing Technology Innovation Center of Hebei Province, Shijiazhuang, China; d Hebei Higher Education Institute Applied Technology Research Center on TCM Formula Preparation, Shijiazhuang, China

**Keywords:** Hyperlipidaemia, lipid accumulation, inflammation

## Abstract

**Context:**

*Swertia mussotii* Franch. (Gentianaceae) is a source of the traditional Tibetan medicine, ZangYinChen, and is used to treat chronic hepatitis and many types of jaundice.

**Objective:**

This study explored the therapeutic effects and mechanism of *S. mussotii* on non-alcoholic fatty liver disease in diet-induced hypercholesterolaemia.

**Materials and methods:**

After a week of adaptive feeding, 32 Sprague-Dawley rats were divided into four groups: (1) Control, (2) Control-S, (3) Model, and (4) Model-S. During the 12 experimental weeks, we established the Model using a high-fat diet. Control-S and Model-S were given 1.0 g/kg *S. mussotii* water extract via gavage starting in the fifth week until the end of experiment.

**Results:**

When compared with Model rats, the *S. mussotii* water extract led to a reduction in high-density lipoproteins (43.9%) and albumin (13.9%) and a decrease in total cholesterol (54.0%), triglyceride (45.6%), low-density lipoproteins (8.6%), aspartate aminotransferase (11.0%), alanine aminotransferase (15.5%), alkaline phosphatase (19.1%), total protein (6.4%), and glucose (20.8%) in serum. A reduction in three cytokines (IL-1β, IL-6, and TNFα) was detected. Histopathological examination showed that liver steatosis was significantly relieved in *S. mussotii*-treated high-fat diet rats. *S. mussotii* also caused a downregulation in the expression of TLR4 (43.2%), MyD88 (33.3%), and a decrease in phosphorylation of NF-κB.

**Discussion and conclusions:**

Our findings indicate that *S. mussotii* may act as a potential anti-inflammation drug via inhibition of the TLR4/MyD88/NF-κB pathway. Further *in vivo* and *in vitro* studies are needed to validate its potential in clinical medicine.

## Introduction

Non-alcoholic fatty liver disease (NAFLD) is defined as a significant accumulation of liver lipids in the absence of a clear cause of any type of liver disease. In recent years, NAFLD has rapidly become the most common cause of chronic liver disease worldwide, and its occurrence has begun to show a trend in younger people (Shimizu et al. 2019; Nasiri-Ansari et al. 2021; Zamani-Garmsiri et al. 2021). Hyperlipidaemia caused by high fat and high sugar diets, unhealthy work style, poor physical exercise levels, fat, and other factors is the main risk factor for NAFLD (Fouad et al. 2021). The prevalence of obesity, diabetes, and/or metabolic syndrome is estimated to be 70%–90%. Most of these patients have hyperlipidaemia (Lu et al. 2021). It is necessary to pay enough attention to the problem of NAFLD and take active measures of prevention and treatment.

The pathogenesis of NAFLD is not entirely clear, but studies have shown that it is closely related to toll-like receptor 4, myeloid differentiation factor 88, and nuclear factor kappa-B (TLR4, MyD88, and NF-κB, respectively) as described by several groups of researchers (Hu et al. 2020; Liu et al. 2020; Lwin et al. 2021). Usually, NF-κB and the protein inhibitor of nuclear factor kappa-B (IκB) exist as a complex and are inactive. Lipopolysaccharide or free fatty acids activate TLR4 and combine with MyD88 to form a complex. The complex leads to activation of IκB phosphorylation of NF-κB after which the NF transport into the nucleus, binds with the related genes, and then regulates their transcription (Shin et al. 2019). Modern studies have investigated different types of drugs, such as *Cassia* glycosides, astragaloside IV, and others, all of which can produce an obvious improvement in liver function, which is related to reductions in the expression levels of TLR4 and NF-κB in liver thus leading to a reduction in blood lipids in NAFLD rats (Zhang et al. 2016; Kang et al. 2017; Lin et al. 2019).

*Swertia mussotii* Franch. (Gentianaceae) is a source of the Tibetan medicine, ZangYinChen, which is so precious that it was named one of the ‘eight treasures’ (Tian et al. 2014). It has been used in Tibetan medicine for 1400 years and presently has important is used for treating acute and chronic hepatitis and all types of jaundice (Yun and Chen 2020). Modern pharmacological investigations have shown that various extracts of *S. mussotii* can ameliorate hyperlipidaemia in rats with experimentally-induced hyperlipidaemia, repair liver fibrillation, and alleviate the damage due to immunological liver injury (Chai et al. 2015; Feng et al. 2015; Wu et al. 2017; Zhang et al. 2021). However, the actions of *S. mussotii* in NAFLD under conditions of hyperlipidaemia are not clear. In the present study, we explored the potential mechanism of *S. mussotii* in mitigating hepatic damage and inflammation in diet-induced NAFLD rats.

## Materials and methods

**Figure 1.**
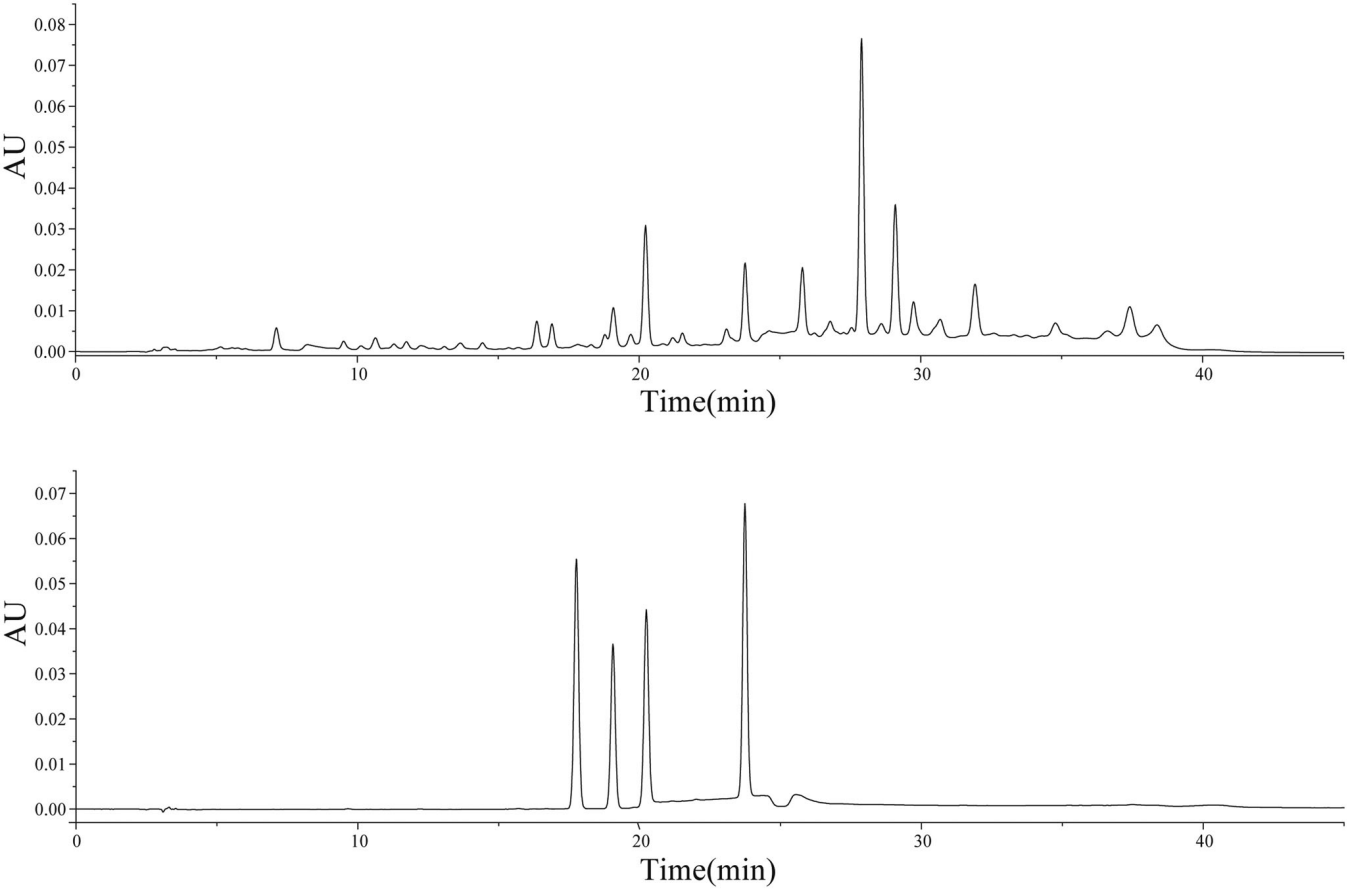
HPLC chromatograms of standard reference xanthones (A) and those from *S. mussotii* Franch (B) are shown, respectively. 1. Sweroside; 2. Gentiopicroside; 3. Swertimarin; 4. Mangiferin.

*S. mussotii* was purchased from the Gerenlugonglong, Zhiduo county, Yushu Tibetan Autonomous Prefecture, QingHai districts (95.85, 33.86) and was identified and authenticated by Professor Yu-Ping Yan (medicinal plants, College of Pharmacy, Hebei University of Chinese Medicine). *S. mussotii* herbarium specimens are stored in the warehouse on the first floor of the scientific Research Building of Hebei University of Traditional Chinese Medicine. The plants were air-dried and then chopped. Fifty grams of *S. mussotii* were soaked in 500 mL distilled water for 1 h, boiled in two batches, and combined twice with the filtrate. The mixture was concentrated, and a suspension of *S. mussotii* with a concentration of 0.10 g/mL was obtained. The *S. mussotii* extracts were evaluated by high performance liquid chromatography (HPLC) analysis and the presence of three iridoid glycosides and Mangiferin were identified (Figure 1). HPLC analysis was performed by Waters 2695 with a Waters 2489 ultraviolet detector on a Zorbax C18 SB-AQ (4.6mm × 250 mm, 5 μm) maintained at 30 °C. The mobile phase, A, consisted of 0.1% formic acid, and the mobile phase B consisted of methyl alcohol. The total run time was 40 min. The injection volume was 10 mL, and the flow rate was set at 1.0 mL/min. Detection wavelength was set at 240 nm.

### Animals and experimental design

We followed the method of Si et al. (2021) for which 32 specific pathogen-free male Sprague Dawley rats weighing 160–180 g were purchased from Beijing Vital River Laboratory Animal Technology Co., Ltd. and were adaptively fed at room temperature for 1 week. Rats were randomized into four groups, each containing eight rats: (1) normal diet (Control), (2) normal diet + *S. mussotii* (Control-S), (3) high-fat diet (Model), and (4) high-fat diet + *S. mussotii* (Model-S). The high-fat diet (high-fat feed ratio: 80.4% basic feed +2% cholesterol + 10% lard +0.5% sodium cholate +0.1% propylthiouracil +5% sugar +2% yolk powder) was used to induce hyperlipidaemia. Normal and high-fat feed was provided and prepared by Hebei Medical University. After searching the literature, we designed a 1.0 g/kg *S. mussotii* solution (Ren et al. 2020). After four weeks of modelling, the rats in the Control-S and Model-S groups were given 1.0 g/mL *S. mussotii*, and the other groups were treated with the same amount of physiological saline for another 8 weeks.

At the end of the experiments, all rats were only provided with water for the last 12 h. All rats were anaesthetized with 0.4 mL/kg 3% pentobarbital (Sigma-Aldrich Corporation, USA). Blood was collected from the femoral artery, and serum was separated by centrifugation at 3500 rpm for 15 min and stored in a refrigerator at −20 °C to be used in further analyses. The liver was weighed and fixed in 10% formalin for histopathological studies while the rest was stored at −80 °C.

All procedures, protocols, treatments, sampling, and euthanasia were approved and supervised by the Ethics Committee of Hebei University of Chinese Medicine (approval no. DWLL2018017).

### Biochemical index test

Total cholesterol and triglyceride contents (TC and TG, respectively) were measured using the single reagent method. Low- and high-density lipoproteins (LDL and HDL, respectively), aspartate aminotransferase (AST), alanine aminotransferase (ALT), alkaline phosphatase (ALP), total protein (TP), albumin (ALB), and glucose (GLU) in serum and interleukins-1β and −6 and tumour necrosis factors alpha (IL-1β, and −6 and TNFα, respectively) in livers were measured according to the manufacturer’s instructions.

### Histopathological examination of the liver

The frozen liver sections (4 μm each section) isolated from each group were fixed in 10% (v/v) formalin in 50 mmol/L potassium phosphate buffer (pH 7.0) for 24 h at 4 °C. The tissues were subsequently embedded in paraffin, cut into 4 μm sections, and stained for 5 min at room temperature with haematoxylin and then 1 min with eosin at room temperature (H&E). Slides were observed and photographed with a Leica DFC 320 digital camera (Leica Microsystems Imaging Solutions Ltd; Cambridge, UK).

### Oil red staining of the liver

The frozen sections prepared in advance and stored at −20 °C were removed from the refrigerator and rewarmed at room temperature for 5–10 min. The dye solution was prepared according to the ratio of storage solution to diluent (5 : 2) and then filtered slowly with filter paper for reserve. Frozen sections were placed in staining solution for 15 min and then rinsed in distilled water at 37 °C for 15 s after which they were re-stained for 5 min and washed for 45 s. Microscopic examinations were performed immediately after the film was sealed.

### Immunohistochemical analysis of IKKβ and IκBα in liver

Each section was dewaxed with a dimethylbenzene gradient and dehydrated through an alcohol gradient. At room temperature, the section was incubated with 3% hydrogen peroxide (H_2_O_2_) for 20 min, avoiding light, and then rinsed three times in phosphate-buffered saline (PBS) for 5 min per wash. The section was blocked with goat serum (cat. no. ZLI-9056; ZSGB-BIO; OriGene Technologies, Inc.) for 30 min at room temperature. The primary antibodies, IKKβ (Bioss, bs-2910R, 1:100) and nuclear factor of kappa light chain polypeptide gene enhancer in B-cells inhibitor alpha ([IκBα]; Bioss, bs-1287R, 1:100), were incubated with the sections overnight at 4 °C. At room temperature, secondary antibodies were added to sections and incubated for 20 min in the dark after which they were rinsed three times in PBS for 3 min per wash. The sections were stained with diaminobenzidine (DAB) reagent, dehydrated with alcohol gradient and DAB, and finally mounted with neutral balsam. The sections were viewed under a light microscope and analysed with Image-Pro Plus 6.0.

### Western blotting for TLR4, MyD88, NF-κB, and p-NF-κB in liver

Liver tissue (50 mg) was finely chopped and added with 400 μL PBS, homogenized, centrifuged to take precipitate and add cytoplasmic protein extraction reagent (R0500), blow and beat for 20 s, ice water bath for 10 min, centrifuged 10 min, supernatant was cytoplasmic protein, precipitate was added nuclear protein extraction reagent (R0500), same as above blow and ice water bath for centrifugation, supernatant was nuclear protein. The protein extract was performed and analysed using a BCA Protein Assay Kit (Beyotime, P0010), separated on a 10% sodium dodecyl sulfate-polyacrylamide gel electrophoresis, and then transferred to polyvinylidene difluoride membranes. Membranes were blocked for 5 h with 5% non-fat dry milk in Tris-buffered saline with Tween 20 and left overnight. The blots were incubated with primary antibodies for TLR4 (Bioss, bs-20594R, 1:500), MyD88 (Bioss, bs-1047R, 1:600), NF-κB (Bioss, bs-20159R, 1:800), and p-NF-κB (Bioss, bs-5662R, 1:600) overnight at 4 °C and then incubated with secondary antibody conjugated to horseradish peroxidase (Biosharp, 1:3000) for 2 h at room temperature. The protein bands were quantified by transmittance densitometry using Image J software. The relative band intensity of the protein was expressed as the ratio of each protein to the reference glyceraldehyde 3-phosphate dehydrogenase.

### Statistical analysis

All statistical analyses were completed using IBM SPSS 22.0 software (IBM SPSS, Inc., Chicago, IL, USA). The data are presented as mean values ± standard deviations (SD). Differences among the four groups were assessed using one-way analysis of variance followed by Tukey’s test and were considered statistically significant at *p* < .05.

## Results

### Effects of *S. mussotii* on serum lipids

**Figure 2.**
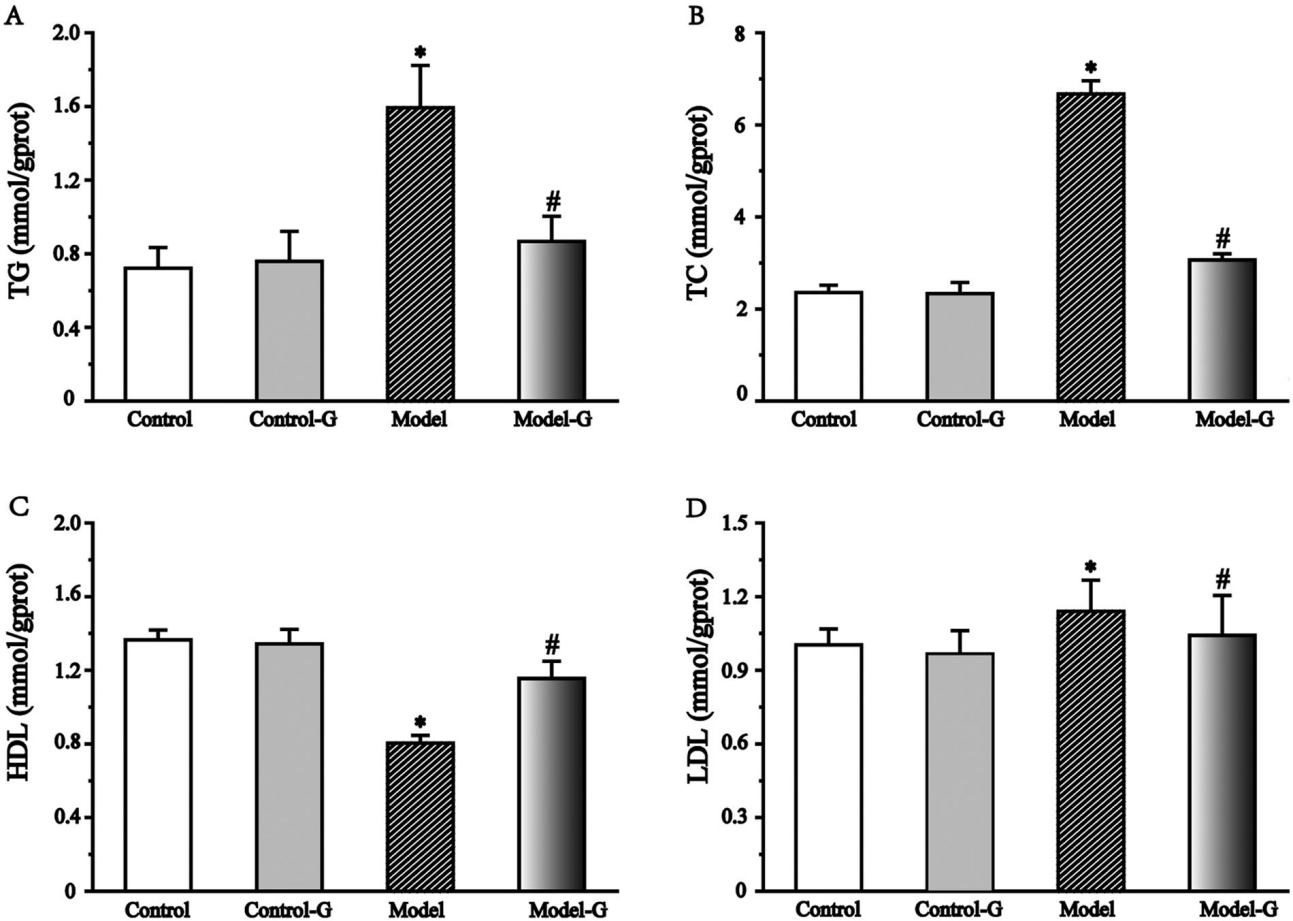
Effects of *S. mussotii* on serum lipid levels. Serum (A) TG, (B) TC, (C) HDL, and (D) LDL levels following treatment with *S. mussotii*. Data are presented as the mean ± SD. *p < 0.05 vs. Control group; ^#^*p* < 0.05 vs. Model group (*n* = 8 per group).

When compared with the Control group, the TG, TC, and LDL levels of the Model and Model-S groups showed a significant increase (120.8%, 182.8%, and 13.6%, respectively), while the HDL level showed a decrease (41.1%) significantly (*p* < 0.05). The four levels of the Model-S group were also extremely different from the Model group in that TG, TC, and LDL levels were reduced (45.6%, 54.0%, and 8.6%, respectively), and HDL had increased (43.9%). The *p* was < .05 as shown in Figure 2.

### Effects of *S. mussotii* on hepatic function levels in serum

**Figure 3.**
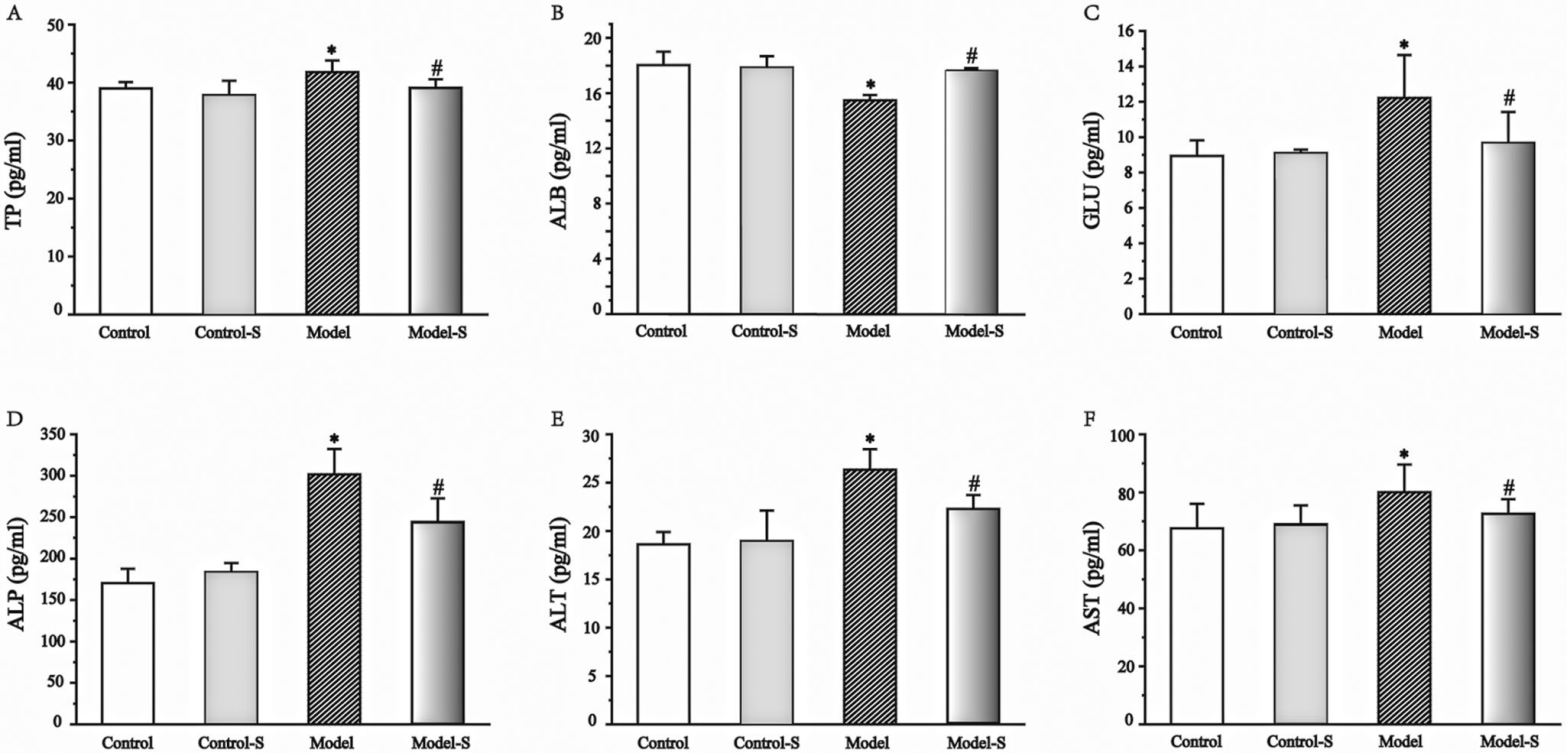
Effects of *S. mussotii* on hepatic function levels in the serum. Serum (A) TP; (B) ALB, (C) GLU, (D) ALP; (E) ALT; and (F) AST levels following treatment with *S. mussotii*. Data are presented as the mean ± SD. *p < 0.05 vs. Control group; ^#^*p* < 0.05 vs. Model group.

Serum levels of GLU, ALP, TP, ALT, and AST, were all higher in the Model group when compared with the Control and Model-S groups (36.8% and 20.8%, 77.1% and 19.1%, 70.9% and 6.4%, 41.5% and 15.5%, and 39.0% and 11.0%, respectively), showing that consumption of *S. mussoti* was effective in treating metabolic complications caused by consumption of a high-fat diet. Meanwhile, there was no statistically significant difference in the measurements of GLU, ALP, TP, ALT, and AST between the Control and Control-S groups (Figure 3).

### Effects of *S. mussotii* on IL-1β, IL-6, and TNFα levels in liver

**Figure 4.**
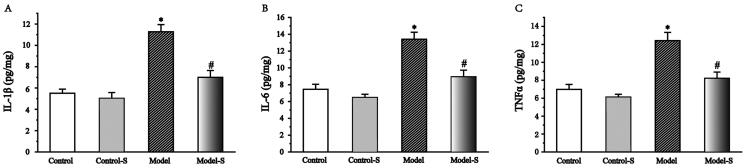
Effects of *S. mussotii* on interleukin-1β and -6 and tumour necrosis alpha (IL-1β and -6 and TNFα levels, respectively) in serum. Serum (A) IL-1β, (B) IL-6, and (C) TNFα levels following treatment with *S. mussotii*. Data are presented as the mean ± SD. *p < 0.05 vs. Control group; ^#^*p* < 0.05 vs. Model group.

The levels of IL-1β and −6 and TNFα levels in the Model group were significantly higher than in the Control (104.5%, 79.8%, 77.8%, respectively) and Model-S (61.0%, 50.0%, 51.0%, respectively) groups (*p* < .05). Meanwhile, no significant differences between the Control and Control-S groups were found. No significant differences between the Control and Control-S groups were found (Figure 4).

### Effects of *S. mussotii* on H&E and oil red staining

**Figure 5.**
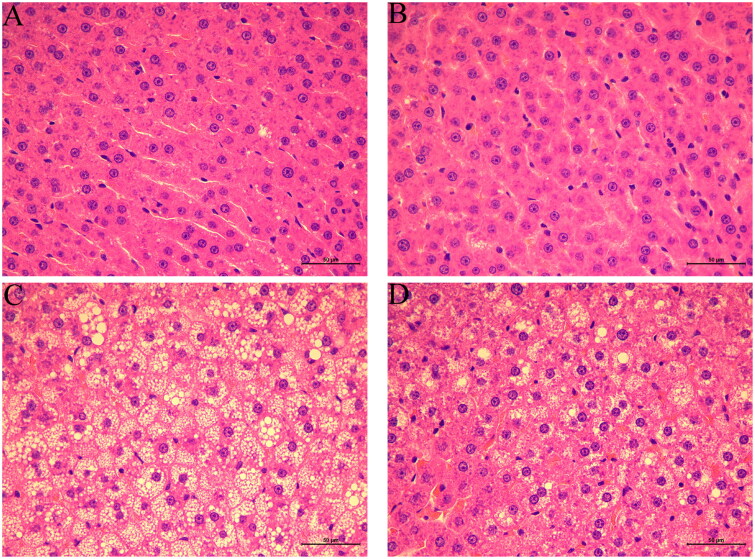
Effects of *S. mussotii* on the histopathological changes in the liver. Liver tissues obtained from the (A) Control, (B) Control-S, (C) Model, and (D) Model-S groups. Scale bar, 50 μm.

**Figure 6.**
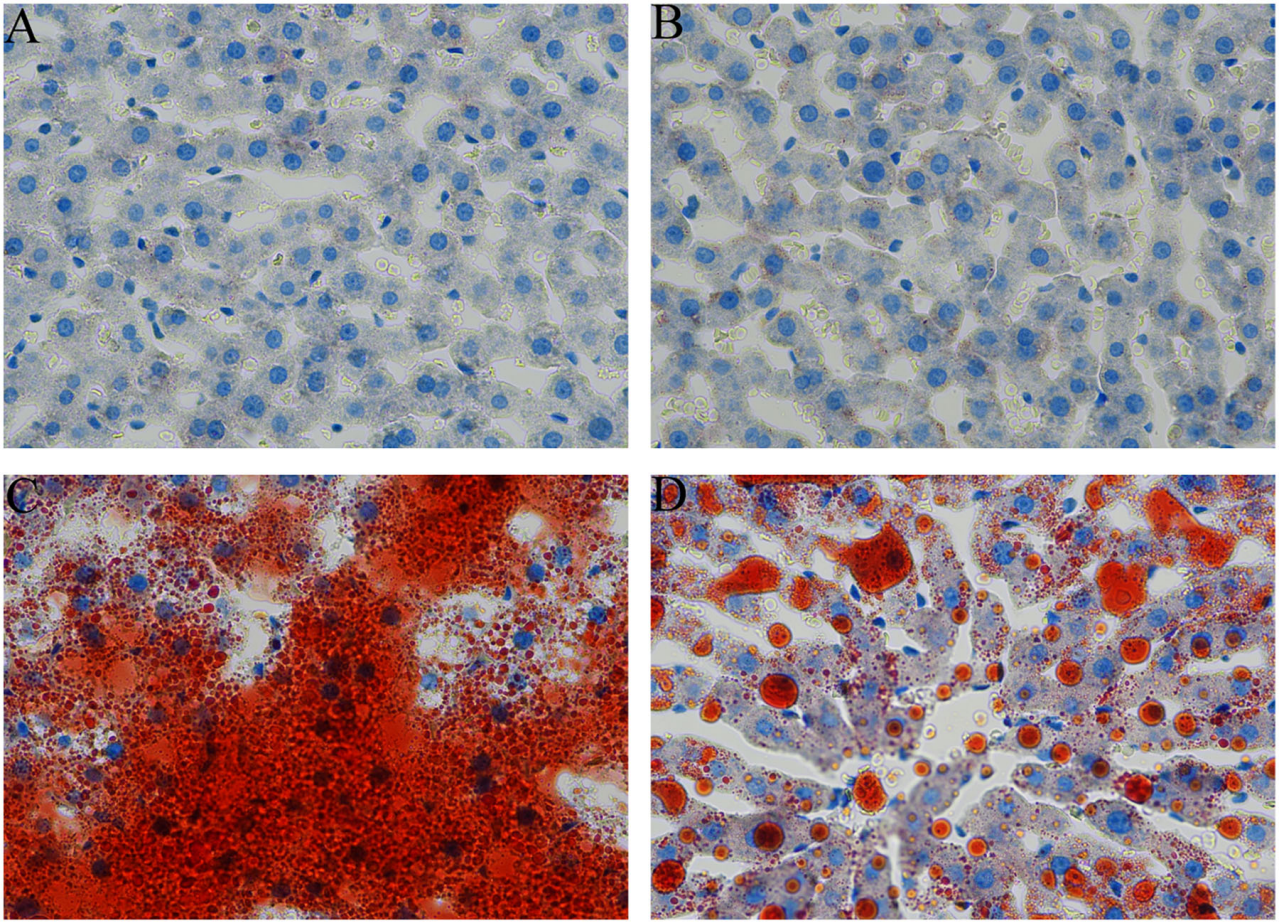
Effects of *S. mussotii* on the oil red O staining changes in the liver. Liver tissues obtained from the (A) Control, (B) Control-S, (C) Model and (D) Model-S groups. Scale bar, 50 μm.

In the Control group, steatosis and necrosis of liver cells were not observed, and no lipid droplets were found in the liver cells. The hepatic sinuses were clearly visible, and the hepatic cords were neatly arranged. Severe cellular steatosis occurred in the livers of the rats in the Model group. The hepatocytes were enlarged, and many vacuolar lipid droplets of different sizes appeared in the cytoplasm, caused by compression of the nucleus to the cell edge. The hepatic sinuses were compressed and narrowed, and the hepatic cord was found to be in a disorderly arrangement. In the Model-S group, the degree of steatosis in liver cells was significantly reduced, and the vacuolisation of lipid droplets in the cytoplasm was reduced; however, large lipid droplets were still visible (Figure 5). Lipid deposition in hepatocytes of the Model group was significantly higher than in the Control group. Lipid deposition in hepatocytes of Model-S was significantly reduced when compared with the Control group (Figure 6).

### Effect of *S. mussotii* on IKKβ and IκBα in liver

**Figure 7.**
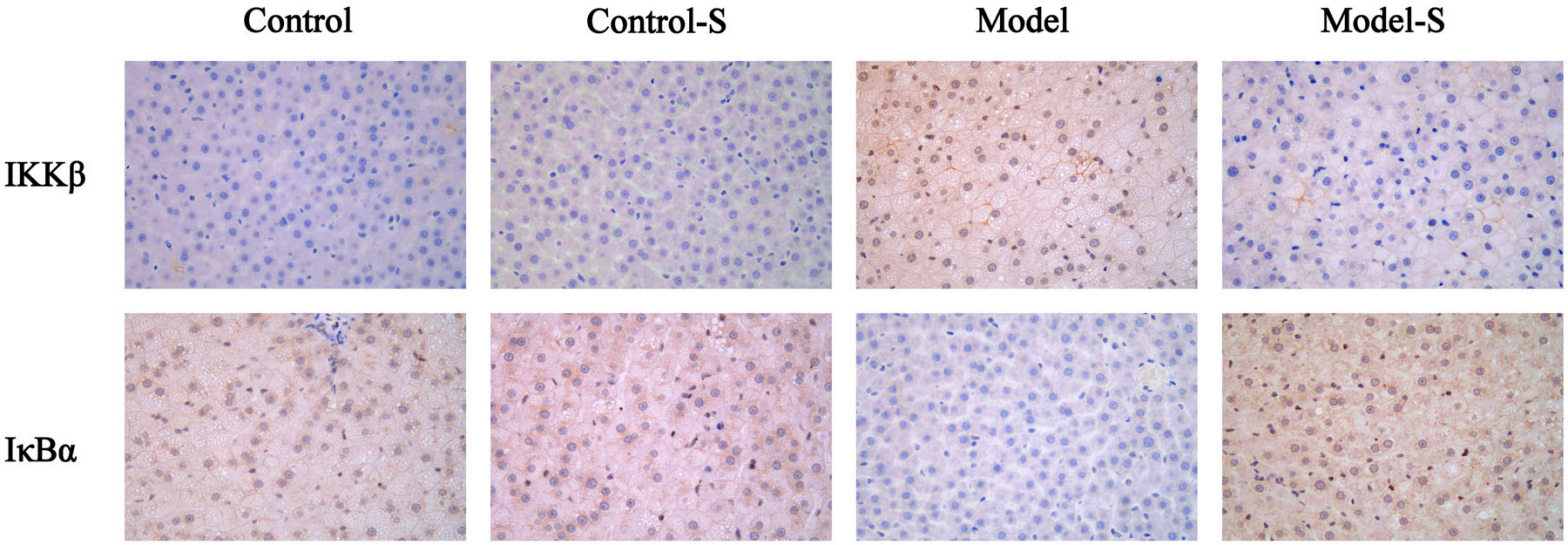
Effects of *S. mussotii* on inhibitor of nuclear factor kappa-B kinase subunit beta (IKKβ) and nuclear factor of kappa light chain polypeptide gene enhance in B-cells inhibitor alpha (IκBα) expression levels in the liver (magnification, ×400).

IKKβ expression levels in the Model group were much higher than the other three groups and the expression levels of IκBα in the Model group were significantly lower than in the others (Figure 7).

### Effect of *S. mussotii* on TLR4/MyD88/NF-κB pathway in liver

**Figure 8.**
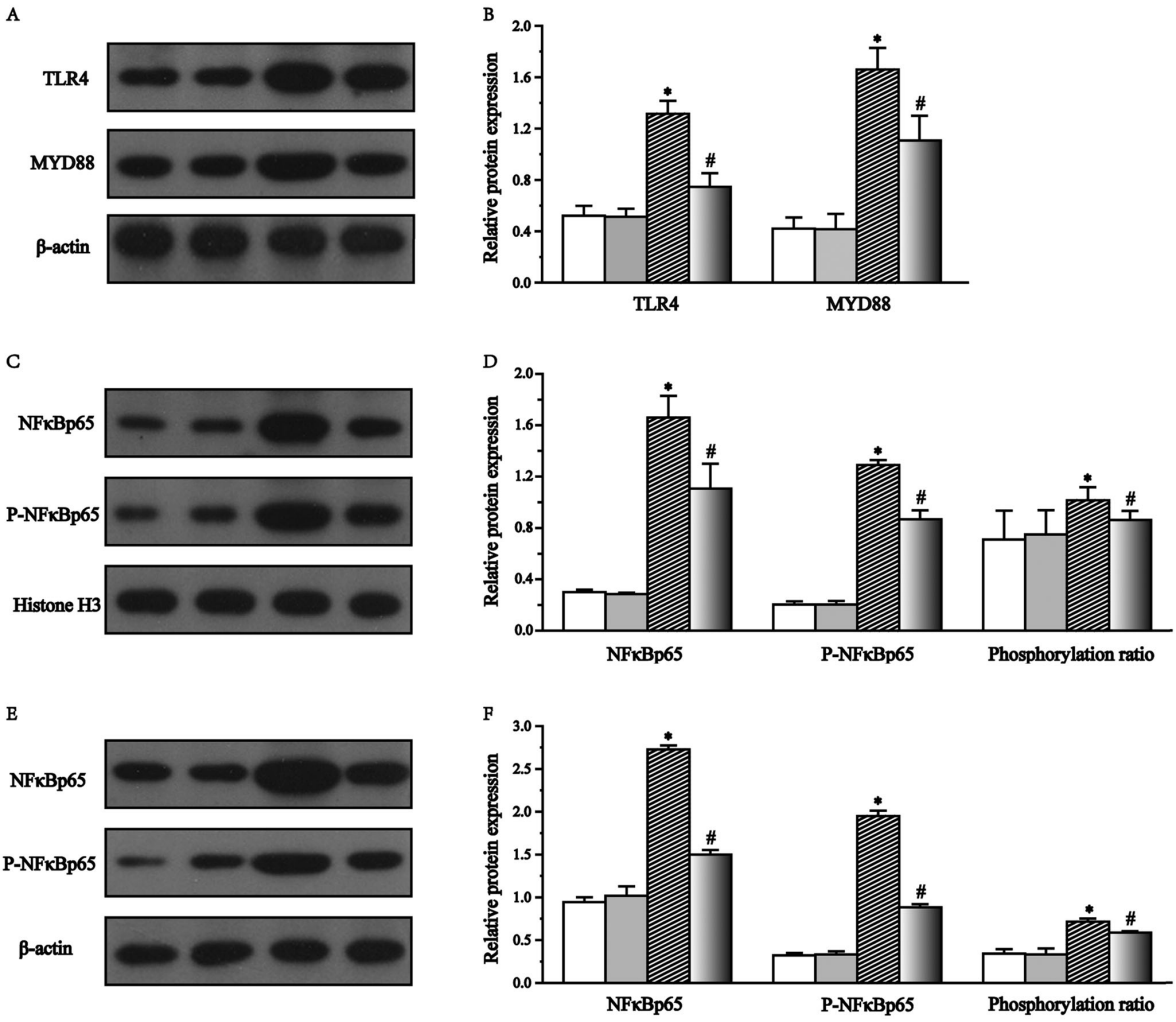
Effects of *S. mussotii* treatment on toll-like receptor 4 and myeloid differentiation factor (TLR4 and MyD88, respectively) in liver and nuclear factor kappa beta and phosphorylated nuclear factor kappa beta (NF-κB and p-NF-κB expression levels, respectively) in the hepatocyte nucleus and cytoplasm. (A) TLR4 and MyD88 typical western blotting bands. (B) Expression of TLR4 and MyD88 in the liver. (C) NF-κB and p-NF-κB typical western bands in hepatocyte nucleus. (D) Expression of NF-κB and p-NF-κB in hepatocyte nucleus. (E) NF-κB and p-NF-κB typical western bands in hepatocyte cytoplasm. (F) Expression of NF-κB and p-NF-κB in hepatocyte cytoplasm. Data are presented as the mean ± SD. *p < 0.05 vs. Control group; ^#^*p* < 0.05 vs. Model group.

TLR4 and MyD88 protein expression levels in the Model group were significantly higher than in the other three groups (*p* < .05). When compared with the Model group, the expression levels of TLR4 and MyD88 had decreased by 43.2% and 33.3%, respectively, in the Model-S (Figure 8(A,B)). Expression and phosphorylation levels of NF-κB significantly increased in both the liver nuclei and cytoplasm of Model rats when compared with the Control rats. Expression and phosphorylation levels of NF-κB were significantly decreased in both liver nuclei (50.0% and 17.9%, respectively) and cytoplasm (81.6% and 21.6%, respectively) of Model-S compared to Model rats (Figure 8(C–F)).

## Discussion

*S. mussotii* appears to be effective for treating liver diseases, such as jaundice, and we propose that *S. mussotii* can be used to treat NAFLD rats under conditions of hyperlipidaemia by regulating the TLR4/MyD88/NF-κB pathway.

The effects of the *S. mussotii* were also evaluated based on serum lipid levels (Ren et al. 2020; Zhang et al. 2021), liver function, and liver levels (Tao et al. 2021) in addition to oil red and H&E staining (Ben Hamad Bouhamed et al. 2019; Yang et al. 2020; Wang et al. 2021). The change in serum lipids levels and oil red staining in the four groups suggest that the NAFLD model of hyperlipidaemia has been established successfully using a high-fat diet and could be effectively treated with *S. mussotii*. When comparing the Model group with the Model-S group, the intrahepatic TC of model-S was noticeably smaller than in the Model group, suggesting that *S. mussotii* can ameliorate hepatic lipid metabolism (Figures 5 and 6). Serum lipids and intrahepatic TC can represent the status of hepatic lipid metabolism. Intra-hepatic TC in the Model and the Model-S groups was effectively reduced by the addition of *S. mussotii* without altering HDL levels, which is a beneficial effect (Figure 2). This result shows that *S. mussotii* can ameliorate hyperlipidaemia as Tsai et al. (2021) suggests in his report. The serum levels of GLU, TP, TLP, ALB, ALT, and AST were all higher in the Model group when compared with the Control, Control-S, and Model-S groups, indicating that the effects of consumption of *S. mussotii*-treated high-fat diets alleviated complications caused by high-fat diets alone (Figure 3). Despite no similarity to the Control group, in the Model-S group, the *S. mussotii* appears to have mitigated this increase since the Model-S group showed lower cholesterol plasma levels when compared with the Model group. The above results describing liver function results show that *S. mussotii* can ameliorate poor liver function caused by NAFLD.

Inflammation plays an important role in NAFLD development and aggravation of *S. mussoii*; moreover, many studies have shown that *S. mussoti* possesses high anti-inflammatory activities (Hassan et al. 2019; Yamamoto et al. 2020). IL-1β and −6 are classic indicators of inflammation, and TNFα is a pro-inflammatory factor produced by macrophages, which can stimulate monocytes and neutrophils to secrete IL-1β and −6 (Chaudhari and Kumar 2020; Fouad et al. 2020). The levels of IL-1β and −6 and TNFα in the Model group were significantly higher than Model-S group suggest that the *S. mussotii* was capable of producing an improvement in the anti-inflammatory liver properties of obese rat (Figure 4). TLR4s play an important role in recognising endogenous and exogenous receptors and inducing the expression of inflammatory factors and are an important part of the body’s immune system (Hassan et al. 2019). TLR4 can be expressed by all liver cells. TLR4 expression increases when liver cells are injured after which it binds to theMyD88 receptor to activate the IL-1β receptor-related kinase family (Liu et al. 2020; Khan et al. 2021; Lwin et al. 2021; ). When compared with the Model group, the expression levels of TLR4 and MyD88 were higher than in the Model-S group. NF-κB is an important transcription factor that controls the expression of inflammatory cytokines and requires phosphorylation and transfer to the nucleus before it can become functional (Ding et al. 2019; Akhtar et al. 2020). The levels of NF-κB and p-NF-κB and the proportion of phosphorylation in the nucleus and cytoplasm significantly decreased (Figure 8). At the same time, the expression levels of IKK and IκB, which regulates NF-κB in the treatment group, were also significantly lower than that in the model group (Figure 7). The above results suggest that *S. mussoti* may inhibit the expression of the TLR4/MyD88/NF-κB pathway in this study.

## Conclusions

*S. mussotii* has positive therapeutic effects on hyperlipidaemia and high-fat diet-induced NAFLD. Such effects may occur via the inhibition of the TLR4/MyD88/NF-κB pathway. Collectively, the results suggest that *S. mussotii* can provide a new mode for treatment of fatty liver diseases.

## Data Availability

The datasets generated and/or analysed during the present study are available from the corresponding author upon reasonable request.
